# Concomitant Hydatid Disease of the Lower Extremity and Retroperitoneum: A Case Report of Two Rare Locations

**DOI:** 10.7759/cureus.115647

**Published:** 2026-09-02

**Authors:** Kenza Berrada, Anas Orgi, Omar Amriss, Nadia Moussali, Naima El Benna

**Affiliations:** 1 Service de Radiologie de l’Hôpital 20 Août 1953, Centre Hospitalier Universitaire Ibn Rochd, Casablanca, MAR

**Keywords:** ct scan, cyst hydatid, cystic retroperitoneal mass, leg soft-tissue mass, mri, muskuloskeletal mri, ultrasound scan

## Abstract

Cystic echinococcosis is an endemic parasitic disease in Morocco, characterized by cystic lesions, most commonly in the liver or lungs, with lesions in other locations being less common. We report the case of a 38-year-old woman presenting with chronic pain and swelling in her left thigh. An ultrasound of the thigh revealed a multilocular mass located on the posteromedial aspect of the thigh. An MRI of the left thigh showed a multilocular subfascial cystic mass containing multiple fluid-filled vesicles and a sinuous hypointense membrane (serpent sign). An MRI of the thoracolumbar spine and an abdominal CT scan revealed additional lesions in the left psoas muscle and the left retroperitoneal space, in close contact with the kidney. Imaging showed no liver or lung lesions. The patient underwent surgery. The pathology report confirmed that the cystic lesions were of hydatid origin. This case underscores the importance of considering hydatid disease when evaluating cystic masses in soft tissues or the retroperitoneum, even in the absence of liver or lung involvement.

## Introduction

Cystic echinococcosis is a parasitic infection caused by *Echinococcus granulosus*. It is endemic in several countries around the world, including Morocco [1]. The disease manifests as cystic lesions that vary in appearance depending on the stage of infection, with the liver being the most commonly affected organ, followed by the lungs. Retroperitoneal and soft-tissue locations remain rare, particularly in the absence of associated liver or lung involvement. Imaging plays a crucial role in the diagnosis of hydatid cysts, particularly in unusual locations, to prevent potential complications from iatrogenic rupture [2]. This case illustrates a unique presentation involving two distinct, highly unusual sites - the retroperitoneum and soft tissues - occurring simultaneously without any involvement of the primary target organs in a 38-year-old female patient.

## Case presentation

We report a case of a 38-year-old woman presenting with chronic pain and swelling of her left thigh. She had no other significant history (no inflammatory signs, trauma, or prior surgery). An ultrasound was initially performed, revealing a multilocular mass on the posteromedial side of the thigh, with no color Doppler flow. Upon re-evaluating the patient's history, there was evidence of early contact with stray dogs in a rural area where she previously resided and regularly visited. An MRI of the left thigh was performed, revealing a multilocular subfascial cystic mass in its posteromedial compartment, which contained multiple vesicles with marked T2 hyperintensity and T1 hypointensity, with contents showing slight T1 hyperintensity and intermediate T2 signal, as well as a linear, serpentine hypointense membrane, depicting the “serpent sign” (Figure 1).

**Figure 1:**
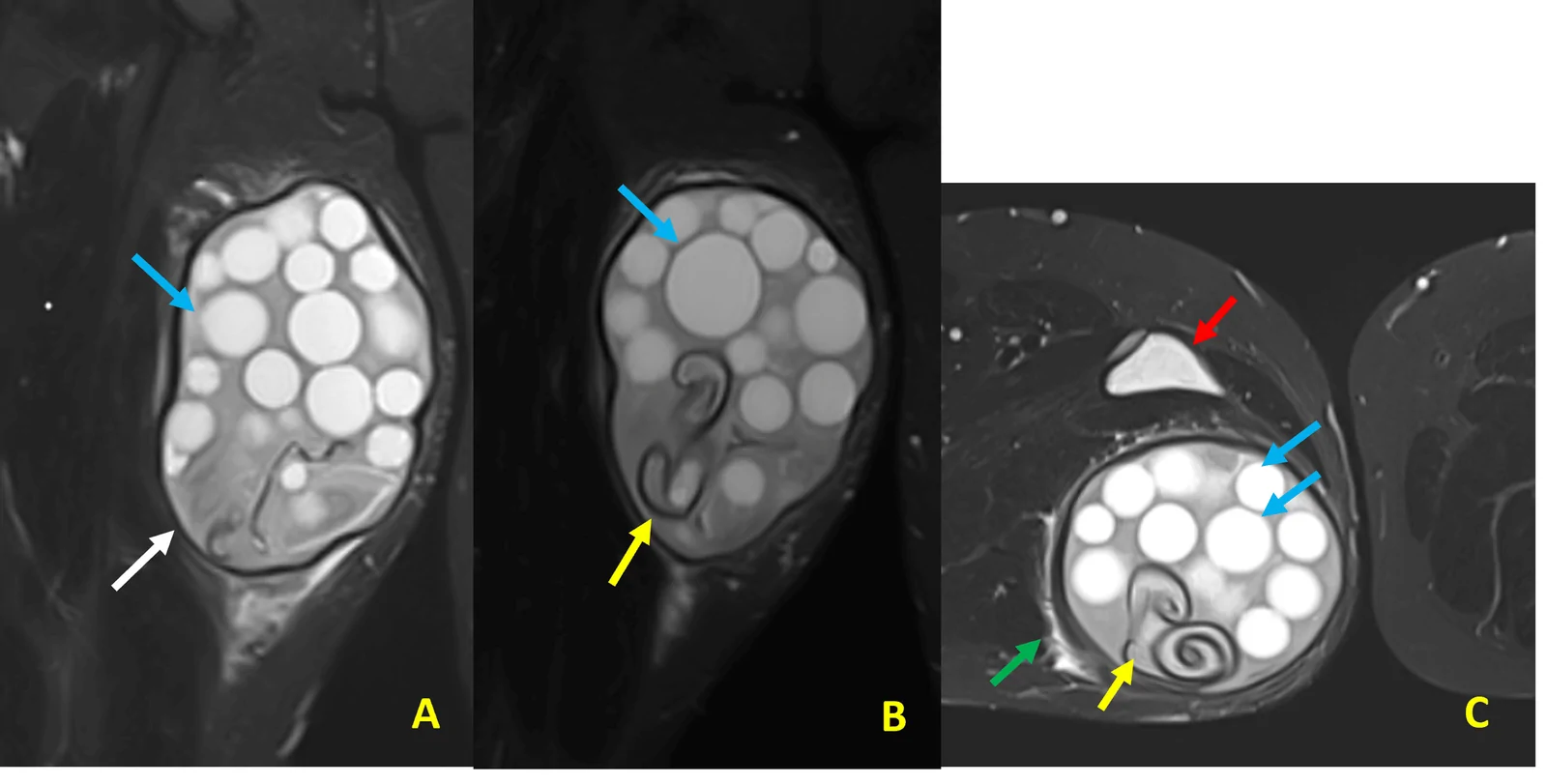
MRI of the left thigh in coronal and axial STIR sequences Coronal (A, B) and axial (C) STIR MRI images of the left thigh. A well-circumscribed, rounded, multicystic lesion is visible in the medial and posterior muscular compartments, extending into the posterior subcutaneous soft tissues. Note the surrounding T2-hypointense rim (white arrow). It contains multiple daughter cysts (blue arrows) and a serpiginous formation with T2-hyposignal intensity forming the serpent sign (yellow arrow), immersed in a T2-hyperintense fluid. There was edematous infiltration of the adjacent subcutaneous soft tissues (green arrow). Another, more anterior loculated cystic mass was found between the adductor longus muscle and the gracilis muscle (red arrow). STIR, short tau inversion recovery

This mass appeared to arise from the adductor magnus muscle, extending between the adductor magnus and the adductor longus muscles, and coming into close contact with the gracilis muscle. It was associated with displacement and thinning, along with edematous infiltration of the adjacent subcutaneous soft tissues (Figure 1). Another, more anterior loculated mass was found between the long adductor muscle and the gracilis muscle; it was oblong and smaller in size and resembled the signal characteristics of the first mass (Figure 1, red arrow). Similar lesions were detected in the upper sections passing through the pelvis. Investigations were eventually completed by a thoraco-lumbar MRI (T2-weighted images in axial, coronal, and sagittal planes) and a thoraco-abdominal CT scan (with and without contrast, with multi-planar reconstruction). Additional lesions were identified within the left psoas major muscle, demonstrating a heterogeneous isointense signal on T2-weighted imaging and hypointensity on T1-weighted sequences. These lesions exerted a mass effect on the aortic bifurcation and abutted the L4 vertebral body, with no evidence of bone erosion, infiltration, or foraminal extension (Figure 2).

**Figure 2:**
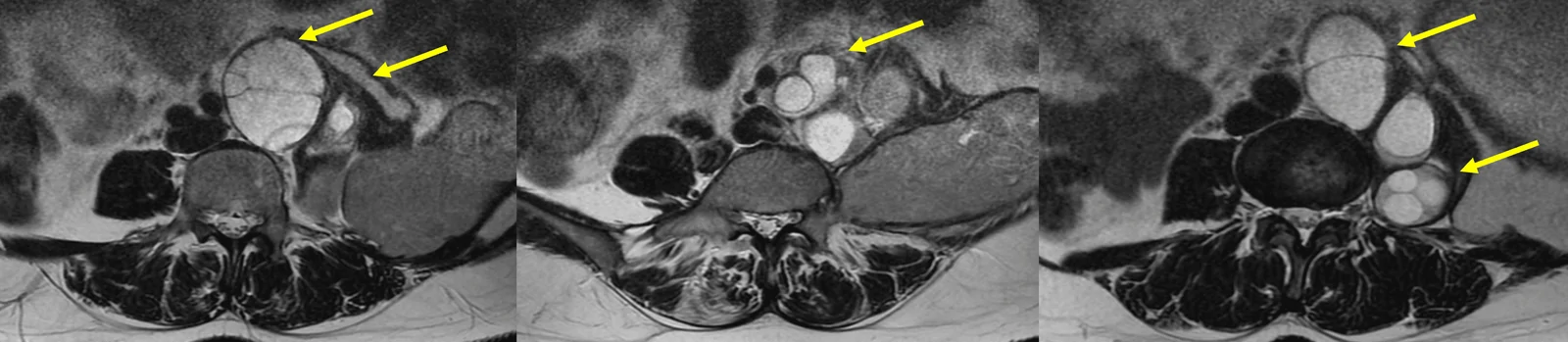
Lumbar MRI in axial T2-weighted sequence showing cystic lesions of the left iliopsoas muscle MRI of the lumbar spine in axial T2-weighted sequence showing multiple cystic lesions (arrows), fairly well-defined, developing within and around the left iliopsoas muscle, the left psoas major and quadratus lumborum, and the inguinal region, with a T2-hyposignal-intensity margin; some of these contain multiple daughter cysts, while others contain heterogeneous, septated fluid or non-septated, remolded fluid, as evidenced by the intermediate signal on T2-weighted images. Some of these formations communicate with one another.

Another cystic mass was located in the iliacus muscle, coming into contact with multiple other masses in the ipsilateral retroperitoneal space, with similar characteristics and calcifications in some areas. The largest of these retroperitoneal masses was multilocular and contained multiple daughter vesicles with T2-hyperintensity, resembling the characteristics of the lesion on the posteromedial aspect of the thigh. On CT imaging, this retroperitoneal mass was well-circumscribed, heterogeneous, containing multiple fluid-filled daughter cysts and a small fatty component. It conformed to the contours of the left kidney (posterolateral aspect), exerting a mass effect on it. The kidney itself harbored an inferior polar cystic formation with exophytic growth and a spontaneously dense wall, coming into close contact with the minimally dilated renal pelvis (Figure 3).

**Figure 3:**
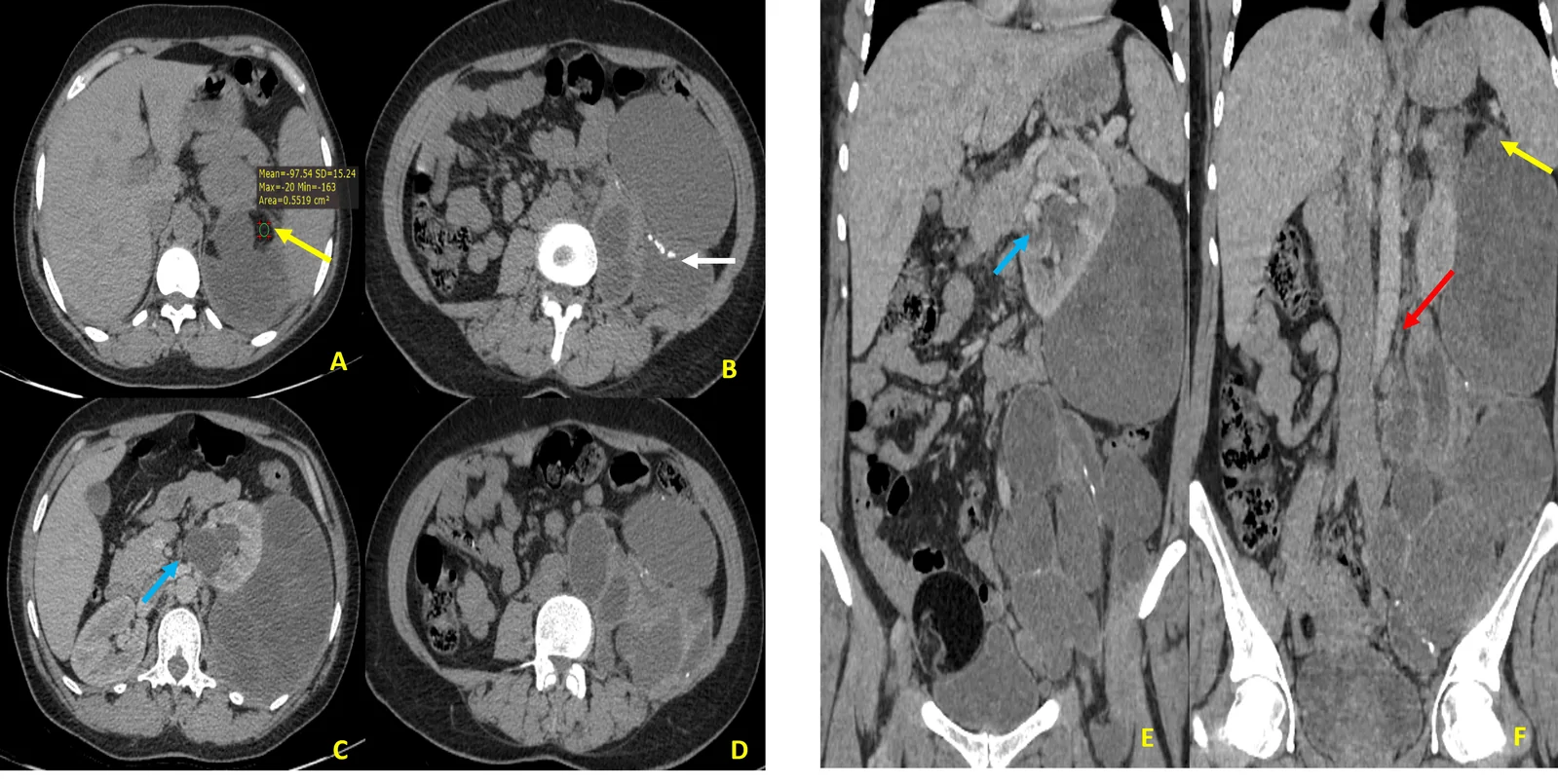
Axial and coronal abdominal CT scans Axial (A to D) and coronal (E, F) CT scan images in the portal phase showing cystic lesions with characteristics similar to those described above, involving the left lumbar psoas, iliopsoas, and quadratus lumborum muscles; some of these lesions appear to communicate with one another and show focal parietal calcifications (white arrow). Note the inward displacement of the aorta, inferior vena cava, and their left bifurcation branches (red arrow). A large, roughly oval mass is noted in the left retroperitoneal space, containing a fluid-fat level (yellow arrow) and multiple daughter cysts, coming into close contact with the left kidney. There was also inferior polar cystic renal formation with exophytic growth and a spontaneously dense wall, coming into close contact with the minimally dilated renal pelvis (blue arrow).

No hepatic or lung lesions were identified. In addition, a right adnexal mass suggestive of an ovarian dermoid cyst was detected; it contained mostly fatty material and showed calcification but was not considered suspicious (likely an incidentaloma) (Figure 4).

**Figure 4:**
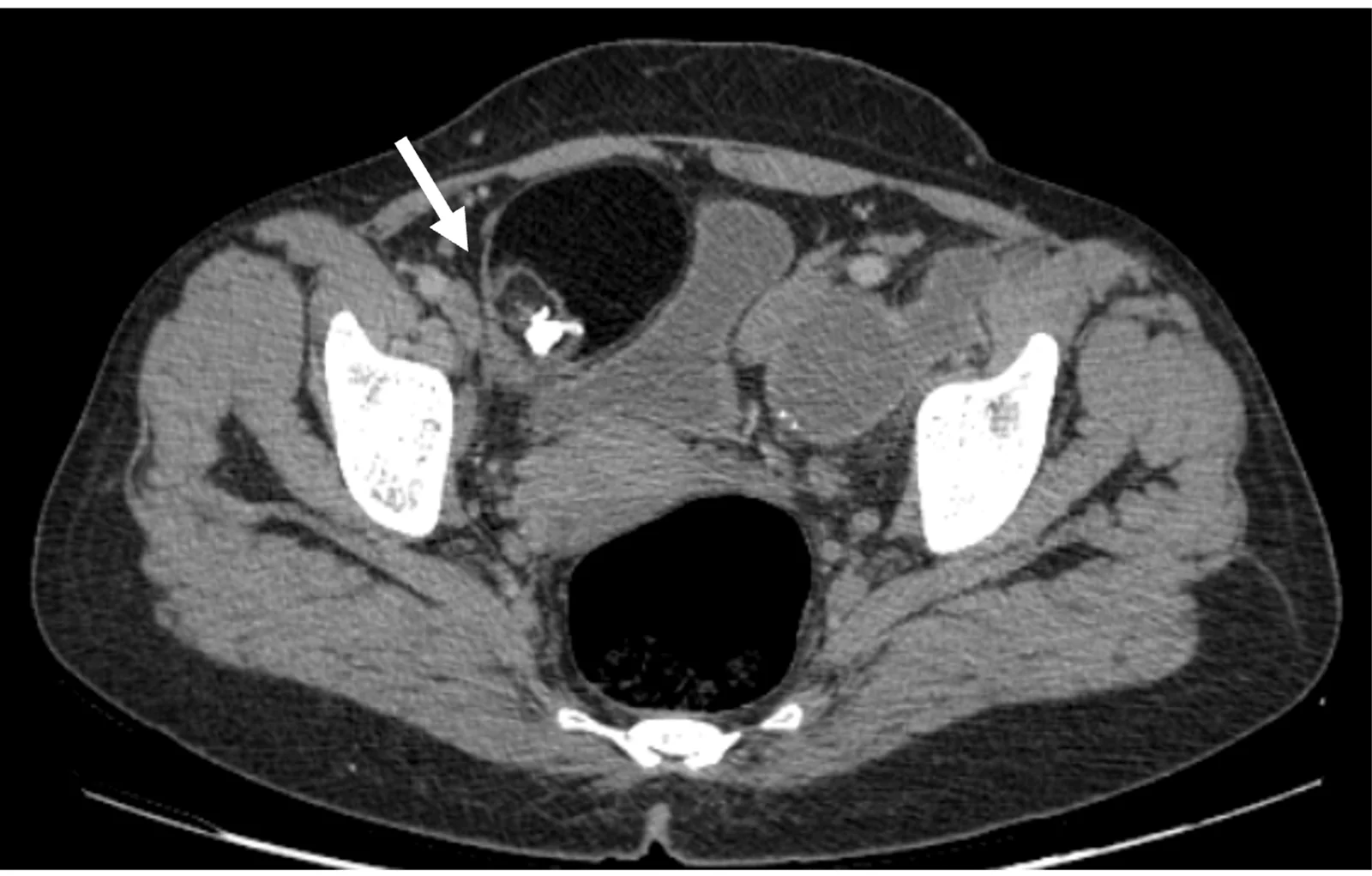
Axial CT scan passing through the pelvis A CT scan showing an axial cross-section of a right adnexal mass suggestive of a dermoid cyst (white arrow), an incidental finding.

With regard to the laboratory results, our patient underwent serological testing for *Echinococcus granulosus* using an indirect hemagglutination test and an ELISA (enzyme-linked immunosorbent assay), both of which returned positive results (1:640 and 20.60, respectively). The eosinophil count was slightly elevated at 700/μL.

The patient was initially treated in the urology department. After ureteral stenting, three purulent collections containing daughter cysts arising from the left psoas muscle, one near the lower pole of the left kidney, and one in the left lateral bladder region were drained (with resection of the protruding dome and irrigation with hydrogen peroxide). Examination of the puncture fluid for parasites also revealed the presence of a few hooklets of *Echinococcus granulosus*. There was no microbiological evidence for a bacterial secondary infection. One month later, she underwent orthopedic surgery to remove the soft-tissue cystic mass from her left thigh. The patient was started on antiparasitic therapy with albendazole. The postoperative course was uneventful, and the pathology report confirmed the hydatid origin of the lesions.

The patient has been followed up and is currently undergoing further evaluation for a suspected recurrence in the retroperitoneal space.

## Discussion

Cystic echinococcosis is a zoonotic parasitic disease caused by *Echinococcus granulosus*. Humans serve as incidental intermediate hosts within the life cycle of *Echinococcus granulosus*, as dogs are the definitive host and sheep the primary intermediate host. Transmission usually occurs via the fecal-oral route (ingestion of eggs from a contaminated environment or direct contact with an infected animal) [3]. Morocco is an endemic area where echinococcosis continues to present a significant public health challenge [1]. The most common sites are the liver, followed by the lungs. Soft-tissue and retroperitoneal involvement are exceedingly rare, particularly in the absence of concomitant hepatic or pulmonary lesions [2]. Clinically, they manifest as slow-growing, indolent masses that frequently mimic benign or malignant soft-tissue neoplasms. Symptoms often remain absent until the cyst achieves significant dimensions [3].

Rare cases of primary hydatid cysts arising within the retroperitoneum, iliopsoas muscle, or other soft tissues have been described in the literature [4-6], but the combination of these locations remains exceptional. In our case, we speculate that the involvement of the soft tissues of the thigh is likely due to spread following a rupture of a retroperitoneal lesion. A few such cases have been reported in the literature [7]. However, as far as our patient is concerned, this remains a theory.

Diagnosis relies on anamnestic data, immunological tests, and imaging. Our patient underwent a hydatid serology test and direct parasitological examination, which came back positive. Serological testing includes ELISA, indirect hemagglutination, Western immunoblots, and latex agglutination assays. It is used to validate imaging findings by detecting antibodies specific to *Echinococcus granulosus *antigens [3,8]. Microscopic examination of the fluid aspirated from the cyst may reveal protoscoleces following staining, confirming the diagnosis [8].

Imaging plays a pivotal role in the diagnosis of hydatid cysts; indeed, unplanned diagnostic puncture is strictly contraindicated, as it exposes the patient to an increased risk of dissemination or anaphylactic shock [9]. Regarding soft tissue localization, ultrasound is the first-line imaging modality for evaluating a mass. It allows for the assessment of the cystic nature of the mass, which is typically multilocular, and the absence of flow on color Doppler. MRI remains the "gold standard" imaging modality due to its inherently high contrast; it is particularly valuable for the evaluation of superficial and deep soft tissues across both large and small fields, as well as the assessment of anatomical relationships. It also facilitates preoperative planning [10].

Regarding retroperitoneal localization, ultrasound remains a readily available first-line imaging modality that can guide the diagnosis. Due to its high spatial resolution, CT scanning is considered the primary method for characterizing the mass (integrity of cyst wall, density, presence of calcifications) and its relationships to surrounding structures, and thus for assessing operability. MRI has a superior soft-tissue resolution, and thus it can be used for some complex cases requiring detailed soft-tissue characterization, but CT remains the modality of choice for precise anatomical and surgical mapping [10].

Our patient had a fat-containing hydatid cyst. It is a documented but rare abnormality, most probably due to the maturation and degeneration of hydatid membranes. Differential diagnosis includes mature cystic teratoma, dermoid cyst, epidermoid cyst, and fat necrosis [11]. The pathology report confirmed the hydatid origin of the retroperitoneal lesions.

Misinterpretation of rare localizations can lead to diagnostic delays, inappropriate treatment, or cyst rupture with potential anaphylaxis. Therefore, it is important to keep in mind hydatid cyst as a differential diagnosis of cystic lesions that grow over time in any region of the body, especially in endemic regions [9].

Treatment modalities include surgical intervention, percutaneous image-guided aspiration (PAIR: puncture, aspiration, injection, reaspiration), and antiparasitic therapy [3]. The pathological examination provides further and definitive confirmation of the diagnosis [10]. Imaging also plays a role in follow-up, particularly during the first two to three years, when recurrence is most likely to occur. Clinical and ultrasound examinations should be done every three to six months and then every year once the situation is stable [8]. CT scan is valuable in the follow-up of retroperitoneal hydatid disease [10].

The study's limitations include the inability to generalize based on a single case, its retrospective nature, the difficulty in accessing various records, and a probable researcher bias.

## Conclusions

Hydatid disease is a zoonosis that can localize to almost every part of the body. This case brings attention to the unusual occurrence of muscular and retroperitoneal hydatid cysts, without involvement of the liver or lungs. Diagnosis relies heavily on imaging. Although significant progress has been made towards combating hydatid disease, it is still a public health issue, particularly in Morocco. It is important to consider the diagnosis of hydatid cyst in any cystic lesion, regardless of its location, especially in endemic regions.
